# Molecular Mechanisms of Glucocorticoid Resistance in Corticotropinomas: New Developments and Drug Targets

**DOI:** 10.3389/fendo.2020.00021

**Published:** 2020-01-29

**Authors:** Denis Ciato, Adriana Albani

**Affiliations:** Medizinische Klinik und Poliklinik IV, Klinikum der Universität, Ludwig-Maximilians-Universität München, Munich, Germany

**Keywords:** glucocorticoid receptor, glucocorticoid resistance, HSP90, silibinin, testicular receptor 4

## Abstract

Cushing's disease is characterized by excessive adrenocorticotropin hormone (ACTH) secretion caused by a corticotroph tumor of the pituitary gland, leading to hypercortisolism and increased morbidity and mortality. The molecular causes of the disease are not completely understood, therefore more research is needed to discover novel molecular targets and more effective treatments. To date, the SSTR-analog pasireotide is the only approved drug for Cushing's Disease treatment that is directly targeting the source of the disease. Targeting directly the activity of glucocorticoid receptor or the factors modulating it might be a new valid option for the medical management of Cushing's disease. Here, we briefly review the molecular mechanisms involved in the glucocorticoid negative feedback and glucocorticoid resistance and examine novel targets and therapies that might effectively restore glucocorticoid sensitivity.

## Introduction

Cushing's disease is a rare endocrine disease resulting from chronic exposure to high cortisol levels as consequence of a pituitary corticotroph tumor that hypersecretes adrenocorticotroph hormone (ACTH). It represents around 80% of all cases of endogenous hypercortisolism (1–3). The management of Cushing's disease relies on the surgical removal of the pituitary tumor with a success rate varying between 79% in pituitary microadenomas and 40% in macroadenomas (4). When the surgery is not feasible or when the disease persists after the surgical intervention the only pituitary-directed drug approved for the treatment of Cushing's disease is the somatostatin analog pasireotide, able to achieve a partial or total disease control in overall 30–40% of patients (5–7). Corticotroph tumors have a monoclonal origin, suggesting that single somatic mutations might be the etiologic mechanism of the disease (8–10). A significant step forward in understanding the pathogenesis of Cushing's disease has been made with the identification of recurrent somatic mutations in the *Ubiquitin-specific-protease 8* gene *(USP8)* in around half of corticotroph tumors (11–14). The current knowledge suggest that *USP8*-mutations result in hyperactivation of the Epidermal growth factor receptor (EGFR) signaling due to increased EGFR deubiquitination with subsequent higher Proopiomelanocortin (*POMC*) promoter activity and increased ACTH secretion. To date, this molecular mechanism does not seem to have an impact on the glucocorticoid resistance itself. Therefore, we are still far from fully discovering the molecular mechanisms leading to the development of partial glucocorticoid resistance, which might be (although being not the only potential pathogenic mechanisms of Cushing's disease) the key target for confirmation of effective therapy. In fact, the partial lack of response to the glucocorticoid negative feedback is used in clinical practice, through the administration of dexamethasone, for the initial diagnosis of Cushing's disease (low dose dexamethasone suppression test, LLDST) and to differentiate between Cushing's disease and ectopic source of ACTH secretion (high-dose HDDST). Whereas, in physiological conditions the administration of low-dose dexamethasone results in suppression of ACTH and cortisol secretion, in patients with Cushing's syndrome it fails to suppress cortisol levels, regardless of the origin of hypercortisolism. Once the diagnosis of Cushing's syndrome is made, the HDSST can be used to distinguish between Cushing's disease and ectopic source of ACTH secretion, based on the evidence that cortisol secretion in corticotroph tumors (and not in ectopic tumors) can undergo partial or complete suppression after high-dose dexamethasone (15–19). Considering the importance of glucocorticoid resistance in corticotroph tumors, a better understanding of the underlying molecular mechanisms could lead to the development of new pituitary target therapies with the aim to restore glucocorticoid sensitivity. In this review, after a short overview of the glucocorticoid negative feedback mechanism, we describe the most recent molecular discoveries in this field.

## The Glucocorticoid Negative Feedback

**Figure 1:**
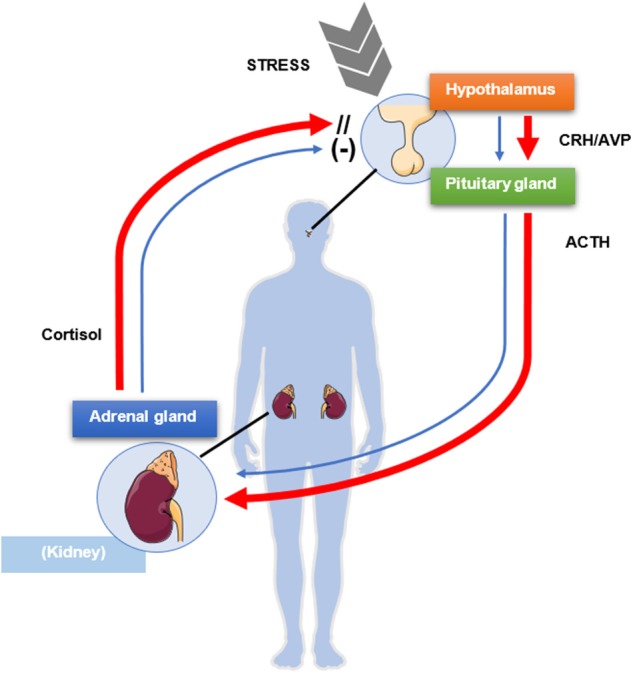
Schematic mechanism of physiologic negative glucocorticoid feedback exerted by cortisol on the pituitary gland and the hypothalamus (blue arrows). A source of stress activates the release of corticotrophin-releasing hormone (CRH) and vasopressin (AVP) from the paraventricular nucleus of the hypothalamus, which in turn stimulate the release of adrenocorticotrophic hormone (ACTH) from the anterior pituitary lobe. ACTH activates synthesis and secretion of cortisol from the adrenal glands, which in turn negatively modulates the release of ACTH from the pituitary gland and of CRH and AVP from the hypothalamus. In presence of Cushing's disease (red arrows), the pituitary gland is only partially sensitive to the inhibitory feedback exerted by cortisol, which in turn is not regulating its own production and secretion, resulting in excessive ACTH and cortisol levels. This figure was modified from Servier Medical Art, licensed under a Creative Common Attribution 3.0 Generic License: http://smart.servier.com/.

The hypothalamic-pituitary-adrenal (HPA) axis is a typical example of endocrine feedback system. The hypothalamus, through the secretion of corticotrophin-releasing hormone (CRH) and to a lesser extent vasopressin, triggers in pituitary corticotroph cells the transcription of *POMC* gene, which leads to the production of the precursor peptide of ACTH (20). In turn, ACTH stimulates the steroidogenesis in adrenocortical cells by binding the melanocortin 2 receptor (MC2R) (21), whereas cortisol and other glucocorticoids exert a negative feedback at hypothalamic and pituitary level, allowing therefore to keep a balance between cortisol requirement and cortisol secretion (22, 23) (Figure 1). Furthermore, cortisol synthesis might be also regulated by itself in an auto-feedback loop within the adrenal gland (24, 25). The physiological glucocorticoid negative feedback on the HPA axis is exerted upon binding to the glucocorticoid receptor (GR), encoded by *NR3C1* gene. The GR belongs to the nuclear receptors family and regulates gene transcription after ligand binding (26). In the absence of ligands, GR is localized in the cytoplasm in a complex with the chaperone heat shock protein 90 (HSP90), other heat shock proteins, and co-chaperones. After binding of the steroidal ligand, GR undergoes a conformational change that promotes its translocation to the nucleus, where it binds to positive or negative glucocorticoid-responsive elements (GRE/nGRE, respectively) to regulate gene transcription.

The transcription of the *POMC* is positively regulated by the activation corticotrophin releasing hormone (CRH) from the hypothalamus, which is balanced by feedback repression in physiological conditions by glucocorticoids (27). In this particular case, three molecules of GR do not bind the nGRE element directly, but through a transrepression mechanism to antagonize the activity of the orphan nuclear receptors Nur77 and Nurr1 (28, 29). Later on, Brahma-related Gene 1 (Brg1, an ATPase subunit of the SWI/SNF complex that is constitutively present on *POMC* promoter) and Histone deacetylase 2 (HDAC2, which recruitment on *POMC* promoter is ligand dependent) were discovered to be essential for the GR transrepression complex to repress *POMC* transcription by blockade of RNA Polymerase II release from the promoter (30).

## Glucocorticoid Resistance

Corticotroph tumors present typically with a compromised response to the glucocorticoid negative feedback which defines a partial glucocorticoid resistance (15) (Figure 1). In fact, whereas the treatment with cortisol led to decreased ACTH levels in human corticotroph tumors in primary cell culture, suggesting the maintenance of a response to the negative glucocorticoid feedback (31), the treatment with dexamethasone led to a lower inhibition of *POMC* transcription and ACTH secretion in corticotroph tumor cells compared with non-tumor pituitary cells, indicating glucocorticoid resistance (32). Multiple factors can influence the response and development of glucocorticoid resistance. These include GR availability, splice variant expression and affinity, and imbalanced GR signaling (33–35). Somatic mutations in the NR3C1 gene have been only rarely reported in corticotroph tumors (17, 36). The genetic screening of 18 corticotroph tumors identified only two polymorphisms of the NR3C1 gene, not correlated with tumor size or clinical presentation, and no NR3C1 mutation (37). In another study the whole exome sequencing of 20 corticotroph tumors revealed two truncating NR3C1 mutations (38). Similarily GR does not seem to be downregulated in corticotroph tumors (39, 40).

Loss of heterozygosity (LOH) at the GR gene locus might be a more frequent and plausible explanation of the relative resistance to the inhibitory feedback of cortisol in corticotroph tumors (17). The potential involvement of other mechanisms, such as the ones controlling the transcriptional GR activity has been postulated (18, 29). The two regulators of chromatin remodeling part of GR transrepression complex, Brg1 HDAC2, have been found to be downregulated in corticotroph tumors and this could contribute to glucocorticoid resistance compromising the ability of glucocorticoids to repress *POMC* transcription (30, 41, 42). At the pre-receptor level, the deregulation of cortisol metabolism could also play a role in the glucocorticoid resistance. High levels of 11-β-hydroxysteroid dehydrogenase 2 (11β-HSD2), converting cortisol to the inactive cortisone, are reported in corticotroph tumors but not in normal corticotroph cells (40, 43).

## Targeting Glucocorticoid Resistance In Cushing'S Disease

### Heat Shock Protein 90

HSP90 is a ubiquitous molecular chaperone that modulates the maturation of more than 200 client proteins, many of which playing a role in cell signaling, response to stress, or modulating cancer and disease progression (44, 45). This task is accomplished with other co-chaperones and proteins that altogether form a complex named the HSP90 chaperone complex, which includes HSP70, HSP40, HSF1, p23, Hip, Hop, FKBP51, and FKBP52 (44). GR is one of HSP90 clients and their interaction is essential for GR ligand binding and activation (46). The affinity of GR to ligands depends crucially on its proper folding. The default, low affinity state of GR has a closed steroid-binding pocket. Only after a radical conformational change, brought about by the chaperone HSP90, the binding pocket opens, allowing mature GR to bind steroids with high affinity. As long as GR remains bound to the HSP90 chaperone complex, the receptor can cycle from a low affinity, partially unfolded state, to a high affinity, fully mature conformation, and then back to its unfolded state (44, 46).

HSP90 has been reported to be overexpressed in several types of cancer in comparison to the normal tissue counterpart, due to increased intracellular stress secondary to hypoxia and acidosis during tumor development (47). In corticotroph tumors HSP90 is overexpressed compared with non-functioning pituitary tumors and normal anterior pituitary (48). In this context, GR would be retained at a higher degree in the cytoplasm by the HSP90 chaperone complex, leading to partial glucocorticoid resistance (48).

Due to the progress in clinical evaluation of targeting HSP90 in cancer, several types of inhibitors are available, which are inhibiting the activity of the N-terminal or the C-terminal portion of the protein (49), both domains that are fundamental for the maturation process of GR before release form the chaperone complex (48). Treatment of corticotroph tumors with silibinin, a C-terminal inhibitor of HSP90, led to enhanced GR transcriptional activity and decreased ACTH production in AtT-20 cells and primary cultures from human corticotroph tumors, and ameliorated Cushing's disease symptoms in an allograft mouse model, with partial restore of glucocorticoid sensitivity. These effects were explained by the trigger of GR release from the HSP90 complex in a fully mature state, and the consequential increased activated GR transcriptional activity in the nucleus to repress *POMC* transcription (48). Its favorable safety profile (classically used for the treatment of toxic liver damage) (50) and its potential chemotherapeutic action (51) suggest silibinin as promising effective drug for the management of Cushing's disease (Table 1). Clinical studies with an improved formulation of silibinin for better delivery into the circulation are now being planned (52).

**Table 1:** Schematic representation of the characteristics of the most recently developed options to restore of glucocorticoid sensitivity on Cushing's Disease.

**Target**	**Role at molecular level**	**Status in Cushing's disease vs. normal**	**Tested compound**	**Molecular mechanism**	**Therapeutical effects**
HSP90	Molecular chaperone	Overexpressed	Silibnin	Inhibition of C terminal domain of the protein causing increased release of mature GR	Inhibition of POMC transcription and ACTH secretion
TR4	Transcriptional factor	Overexpressed	MEK-162	Inhibition of MEK phosphorilating activity	Inhibition of POMC transcription an ACTH secretion

### Testicular Orphan Nuclear Receptor 4

The testicular receptor 4 (TR4 nuclear receptor subfamily 2 group C member 2, also known as NR2C2) belongs to the nuclear receptor superfamily and regulates gene transcription in multiple cellular processes including spermatogenesis, lipoprotein regulation, and central nervous system development (53). Analysis from paraffin-embedded tissues showed that TR4 was found to be overexpressed in human corticotroph tumors with marked localization into the nucleus (54). TR4 overexpression *in vitro* resulted in increased cellular proliferation, tumor invasiveness, *POMC* transcription and ACTH secretion (54). A direct binding site for TR4 has been identified in the *POMC* promoter supporting a direct role of TR4 in the regulation of *POMC* transcription (54). These findings were additionally confirmed *in vivo* by inoculating in nude mice TR4 overexpressing or T4-silencing stable clones from AtT20 cells (54).

Furthermore, co-immunoprecipitation analysis showed that TR4 interacts directly with GR, upon binding its N-terminal domain, and therefore impairing the GR binding with the *POMC* promoter (55). Since TR4 effect on *POMC* promoter was enhanced through its phosphorylation by the mitogen-activated protein kinase/extracellular signal-regulated kinase (MAPK/ERK) pathway (55), the role of the treatment *in vitro* and *in vivo* of human and murine corticotroph tumor AtT20 cells with a MEK inhibitor (MEK-162) has been studied, resulting in inhibition of POMC transcription, ACTH secretion and cell proliferation (56). TR4 knockdown and TR4 overexpression resulted, respectively in a blunted and enhanced inhibitory effect of MEK-162. Chromatin immunoprecipitation analysis showed reduction of TR4 expression and TR4 binding to POMC promoter after MEK-162 (56). All these findings suggest TR4 as a potential therapeutic target to restore the negative glucocorticoid feedback. Having shown acceptable safety profile in previous clinical trials, MEK-162 is another potential good candidate for Cushing's Disease therapy (Table 1). However, it is important to consider that no direct inhibitor of TR4 is available yet, and generally targeting the MEK pathway would influence the activity of multiple factors and not be limited to TR4, therefore potential side effects should be carefully monitored in further studies.

## Conclusions

Some progresses have been made in understanding the molecular mechanism leading to partial glucocorticoid resistance in Cushing's disease, with the discovery of the interaction between glucocorticoid receptors and transcriptional regulators such as TR4 and HSP90. Molecules involved in TR4 signaling pathway and inhibitors of HSP90 are potential promising new drugs for the treatment of Cushing's disease.

## Author Contributions

DC structured the first draft of the manuscript and contributed to the molecular aspects of the disease pathogenesis. AA implemented it and contributed to the state of the art of the topic. Both authors agreed to the final version of the manuscript.

### Conflict of Interest

The authors declare that the research was conducted in the absence of any commercial or financial relationships that could be construed as a potential conflict of interest.
